# The Effect of Sumac Fruit on Serum Lipids and Body Mass Index in Hemodialysis Patients

**DOI:** 10.1155/2022/1687740

**Published:** 2022-10-27

**Authors:** Fereshteh Alahnoori, Tahereh Toulabi, Parastou Kordestani-Moghadam, Fatemeh Jafari Pour, Khatereh Anbari, Iraj Salimikia

**Affiliations:** ^1^Student Research Committee, School of Nursing and Midwifery, Lorestan University of Medical Sciences, Khorramabad, Iran; ^2^Razi Herbal Medicines Research Center, School of Nursing and Midwifery, Lorestan University of Medical Sciences, Khorramabad, Iran; ^3^Social Determinants of Health Research Center, School of Nursing and Midwifery, Lorestan University of Medical Sciences, Khorramabad, Iran; ^4^Department of Nursing, Behbahan Faculty of Medical Sciences, Behbahan, Iran; ^5^Social Determinants of Health Research Center, School of Medicine, Lorestan University of Medical Sciences, Khorramabad, Iran; ^6^Razi Herbal Medicines Research Center, School of Pharmacy, Lorestan University of Medical Sciences, Khorramabad, Iran

## Abstract

**Background:**

Sumac fruit is an antioxidant and reduces serum triglyceride (TG) and cholesterol (CHOL) levels. Therefore, this study aimed to investigate the effect of sumac fruit on serum lipids and body mass index (BMI) of hemodialysis (HD) patients.

**Materials and Methods:**

This triple-blind randomized clinical trial study was conducted for 12 weeks on HD patients. Participants were randomly divided into three groups of 2-gram sumac (*N* = 40), 3-gram sumac (*N* = 40), and placebo (*N* = 40) by nonprobability consecutive sampling and stratified block randomization method based on inclusion criteria. Serum lipids and BMI were measured at the beginning of the study and also at the end of the sixth and the twelfth weeks. The physical activity and 24-hour dietary recall questionnaires were used to collect data.

**Results:**

No significant difference was found between the level of nutrient and micronutrient intake (*P* > 0.05), physical activity (*P*=0.159), and BMI (*P*=0.718) of patients in the three groups before and after the intervention. However, the difference in serum low-density lipoprotein (LDL) levels in each studied group was significant over time (*P* < 0.001). The difference was not statistically significant between the groups before the intervention, 6 and 12 weeks after the intervention (group effect), and between the study groups over time (time-group interaction). No statistically significant difference was observed between the mean levels of TG (*P*=0.875), CHOL level (*P*=0.969), LDL level (*P*=0.998), high-density lipoprotein (HDL) level (*P*=0.136), and BMI (*P*=0.608) in the groups over time.

**Conclusion:**

Consumption of sumac fruit significantly changed the LDL level over time. Although BMI and serum lipids changed in HD patients, these changes were not significant. Future studies are needed to determine the effective dose of sumac and any dose increase should take toxicity into account and consider a larger sample size and longer intervention and follow-up times.

## 1. Introduction

Today, 10% of the world's population suffers from chronic renal failure (CRF) [1]. According to Iran's Center of Transplantation for Specific Diseases Statistics, the number of patients with renal failure is about 25,000. More than 50% of these patients are on hemodialysis (HD) [2]. Up to the end of 2016, 2648,000 patients with renal failure (98%) worldwide were undergoing HD [3]. CRF affects physical function, behavior, mood, cognitive process, socioeconomic status, and especially the quality of life [2, 4]. HD is the most common method of kidney replacement in most patients [5, 6]. It is also associated with high costs, frequent hospital visits, fatigue, a significant reduction in physical ability, stress, anxiety, and low quality of life [5, 7, 8]. Also, one of the causes of dyslipidemia is the withdrawal of *L*-carnitine during HD [9]. HD patients are less active than healthy individuals due to fatigue and other physical and endurance capacity factors. In addition, high CHOL and uric acid levels lead to increased fatigue and decreased daily function in these patients, which may also affect patients' body mass index (BMI) [10]. The results of studies have shown that high BMI is a risk factor for patients with renal failure. BMI is one of the most reliable scales for measuring the probability of being overweight or underweight. BMI is a widely accepted method for defining obesity, which is the division of weight in kilograms by the square of height in centimeters. Globally, it was classified into three groups normal (BMI < 25 kg/m^2^), overweight (BMI ≥ 25–30 kg/m^2^), and obese (BMI ≥30 kg/m^2^) [10, 11].

Renal dysfunction is also associated with many disorders in the metabolism of lipoproteins, which leads to disorders of blood fat and its accumulation. The dysregulated fat metabolism that leads to dyslipidemia is often unrecognized, but almost universal. Dyslipidemia is a common nutritional and metabolic disorder in patients with CRF, which leads to deposition and misplaced distribution of fat in some organs including the kidney, heart, and skeletal muscle, which accelerates inflammation and peripheral diseases. In renal failure, triglycerides (TG), very low-density lipoprotein (VLDL), and low-density lipoprotein (LDL) levels increase and high-density lipoprotein (HDL) decreases [12]. Studies have reported different lipid changes in renal failure [13, 14], with hypertriglyceridemia being the most common of these disorders [15]. In addition to deteriorating renal function, hyperlipidemia increases the incidence of cardiovascular events and mortality in patients with CRF. Early diagnosis and treatment have a supportive effect on the kidneys [16] and significantly reduce the risk of cardiovascular events [17] and the need for coronary artery repair, and mortality [18].

However, therapies such as statin compounds, vitamin B6, and *L*-carnitine are used to treat hyperlipidemia, modulate patients' weight, and reduce their BMI [9, 19]. But the indiscriminate use of synthetic drugs in modern medicine causes dangerous side effects due to their repeated use. Therefore, complementary medicine reduces these complications and prevents the high significant cost of treatment [20]. This type of medicine is often related to diet and lifestyle changes [5, 21]. In this regard, medicinal plants are common treatments [22]. Sumac is one of these medicinal plants, which belongs to the Anacardiaceae family and has a long reputation in traditional medicine for treating intestinal disorders, hepatitis, inflammatory diseases, chronic wounds and acne [23], hypertension [24], cancer, stroke, headache, diabetes mellitus [25], CHOL, and atherosclerosis [26, 27]. It also has antibacterial [28], antioxidant [25, 29], antiischemic, hypouricemic [26], hypoglycemic, and liver-protective effects [30]. Sumac contains phenolic compounds, such as phenolic acids, flavonols, and anthocyanins, which can be a rich source of antioxidants and reduce serum CHOL levels [22]. The hypocholesterolemic effect of sumac reduces the reverse transfer of CHOL, decreases intestinal absorption of CHOL, and even increases bile acid excretion, which lowers CHOL [26]. However, the results of the study conducted by Raufi et al. showed that sumac did not significantly reduce LDL levels compared to lovastatin, so they suggested further studies on this issue [19]. In this regard, Cassel and colleagues recommended nurses and physicians use complementary medicine, herbs, and indigenous therapies [31]. In general, today, due to the very high costs of healthcare and treatment, more attention is being paid to concepts such as proper nutrition, health, physical activity, and preventive medicine such as traditional medicine, especially medicinal plants. On the other hand, due to the increasing number of HD patients and problems such as hyperlipidemia in these patients and the urgent need for treatment, care, and prevention of complications, it is essential to pay close attention to the care of these patients. Few studies have been performed, mainly on animals, and cannot be generalized to humans. Since common synthetic drugs only control one or two factors that affect the disease process, the present study was designed and performed to determine the effect of sumac on serum lipids and BMI of patients undergoing HD.

### 1.1. Study Hypotheses

1. By adjusting the baseline serum lipid values, there is a significant difference between the serum lipid level changes in the groups receiving sumac 2 g, sumac 3 g, and placebo
2. By adjusting the baseline BMI values, there is a significant difference between BMI changes in the groups receiving sumac 2 g, sumac 3 g, and placebo

## 2. Materials and Methods

### 2.1. Study Design and Participants

The present study is a triple-blind randomized controlled clinical trial (IRCT20180407039214N1) with three groups that was conducted between July and August 2018.

### 2.2. Study Setting

The study setting included the educational and medical centers in Khorramabad and Rasoul Akram Hospital in Javanrood, Iran.

### 2.3. Participants

The study population included all patients undergoing HD at the educational and medical centers in Khorramabad and Rasoul Akram Hospital in Javanrood, Iran.

### 2.4. Sample Size Estimation

**Figure 1:**
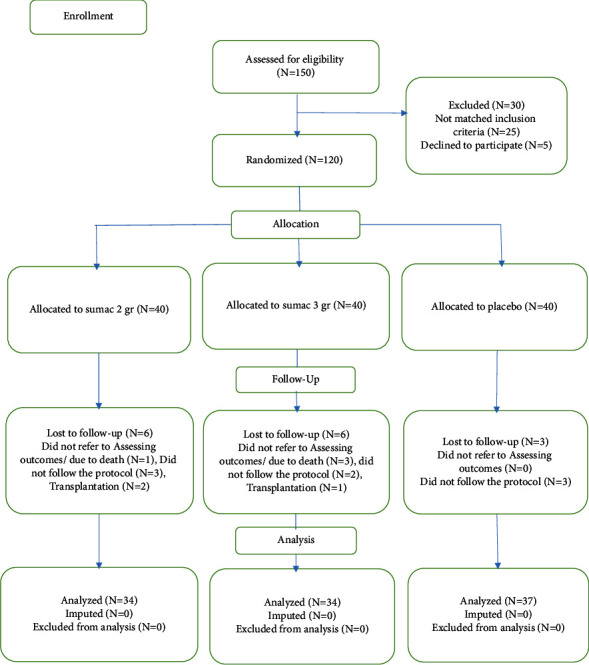
Flowchart of the design, groups, and participants in the study.

The sample size was estimated to be 120 people (*n* = 40 in each group) using the Altman nomogram and considering the test power of 80% and standard deviation of 0.5, taking into account a 10% sample drop. 4 people due to death (1 person in 2 g sumac group and 3 people in 3 g sumac group), 3 people due to transplant (2 people in 2 g sumac group and 1 person in 3 g sumac group) and 8 people due to noncooperation in taking sumac (2 people in 2 g sumac group, 2 people in 3 g sumac group, and 4 people in the placebo group) were excluded from the study and statistical analysis was done on 105 people (Figure 1).

### 2.5. Inclusion Criteria

Inclusion criteria were; willingness to participate in the study with informed consent, having CRF according to the diagnosis of a specialist, being in the age range of 35–70 years, having at least three months of HD three times a week, having the approval of a nephrologist for entering the study (research partner), and being available for assessment three months after the study.

### 2.6. Exclusion Criteria

Exclusion criteria included; death of the patient within the study period, migration, unwillingness of the patient or his/her companion to continue the study, occurrence of allergies during the study period, undergoing kidney transplantation during the study, having cold nature (by patient's statement and examination by a researcher based on a questionnaire), receiving more than one lipid-lowering drug (Gemfibrozil, etc.), having regular and intense exercise, having allergies to sumac fruit and its products, having sensitivity to gluten and celiac disease (with doctor's approval), having mental disorders, experiencing acute pain and illness, having excessive weight loss and morbid obesity (19 ≤ BMI ≤ 40), adherence to weight loss diets under the supervision of a nutritionist, having history of obstructive and inflammatory bowel disease, having severe constipation, being pregnant and lactation, smoking and alcohol consumption, using supplementation and antioxidants during research, participating in other research and programs to prevent cardiovascular disease, and nonregular consumption of sumac and placebo (one day a week according to weekly follow-up).

### 2.7. Drug Preparation and Analysis

Sumac fruit was prepared from the mountainous region of Zagros in western Iran. A botanist identified specimens of sumac fruit. Sumac fruit was packaged in Dana Kasian Pharmaceutical Company in Khorramabad, Iran, in 2-gram and 3-gram sachets. Since the literature review showed that consuming more than 5 g of sumac fruit a day causes gastrointestinal problems, we have used only 2 g and 3 g sachets of sumac in this study after consulting with an herbalist [32].

### 2.8. Blinding

Placebo sachets filled with wheat powder were prepared by the laboratory of the Faculty of Pharmacy, Khorramabad, Iran. Sachets of 2-gram, 3-gram sumac powder, and wheat powder were secretly named sachet *A*, *B* and *C*, respectively, by the research assistant without the researchers' knowledge, participants, and biostatistics experts, and then were provided to the samples.

### 2.9. Randomization

We performed the sampling by a sequential nonprobability method. In this study, subjects were entered from a time point, and sampling was continued until the sample size was sufficient. A stratified block randomization method was used for the random assignment of eligible individuals into three experimental groups. Within each block of 6, two people were assigned to group *A*, two to group *B*, and two to group *C*. We randomly selected a number from the table and, moving from the top to the bottom, numbers 0–24, 25–49, and 75–99 were allocated to group *A*, *B*, and *C*, respectively. After determining the blood lipids and the BMI levels, group randomization was carried out. Also, the effect of BMI was matched with statistical modeling.

### 2.10. Interventions

First, the purpose and method of the study were explained to the patients, and their informed consent was obtained. Eligible patients were matched by gender, age, baseline lipid levels, and medications. Also, the effect of BMI was matched with statistical modeling. Each participant completed a demographic questionnaire containing questions about age, gender, education level, marital status, place of residence, and underlying diseases during the first visit. Also, to assess physical activity, the International Physical Activity Questionnaire (IPAQ), and nutritional behaviors, the 24-hour dietary recall questionnaire was completed by the participant with the help of a researcher. The collected data were analyzed and interpreted by a nutritionist. The weight and height of the participants were measured by the researcher and recorded in the relevant checklists. Individuals' weight was measured and recorded by using a Ska scale with an accuracy of 0.5 kg, and their height was measured using a height gauge installed on the same scale with an accuracy of 0.1. Then, BMI was calculated and recorded based on Adolen's formula (a division of weight in kilograms by height squared in millimeters). Necessary training was given to the subjects one week before the beginning of the study during an introductory session. Subjects were asked to fast for 12 hours before the intervention and at the sixth and twelfth weeks for blood sampling (lipid, TG, serum CHOL, and LDL and HDL levels). On the first day, when the participants received the sachets, they were examined for rash, urticaria, and anaphylactic symptoms and then were recommended to report any problems. Patients were instructed to consume one sachet dissolved in a glass of cool boiled water a day after lunch. Sachets were given to patients at the beginning of the study and in the sixth and twelfth weeks. A physician also visited the patients at the time of dialysis for clinical, laboratory, and possible complications. At the end of each week, patients were reminded how to take the medication and get information about any problems and side effects such as constipation, stomach pain, allergies, etc. They were also reminded that they would be excluded from the study if not take the drugs one day a week.

### 2.11. Outcome Measures and Instruments

#### 2.11.1. Data Collection Form

The demographic information form included personal information (gender, age, residence, education level, and marital status), underlying diseases, medical history of dyslipidemia disorders, and laboratory test results. Intravenous blood samples were taken from the cubital vein at a rate of 5 ccs, then analyzed using a Pars testing kit and BT3000 device. Before the intervention, subjects were instructed to fast for 12 hours, and the venous blood sample was taken by the researcher and placed into sealed tubes without citrate to evaluate lipid, TG, serum CHOL, serum LDL level, and serum HDL level, and then were sent to the laboratory environment of the studied hospitals. The kit and device of all three centers were similar, and all tests were performed automatically. It is worth noting that before taking the blood sample, people were seated to rest for a few minutes. Then, in the shortest possible time, 5 cc of blood was taken from their cubital vein by the researcher and collected in a citrate-free tube in the same package, and then, it was auto-analyzed within one hour using a Pars testing kit BT3000 device.

#### 2.11.2. International Standard Physical Activity Questionnaire

IPAQ was used to assess physical activity. In this questionnaire, the researcher asked about the time of physical activity during the last week. The amount of physical activity is calculated in metabolic equivalent (MET), [33–37]. The amount of MET equals the amount of energy consumed at rest. In this questionnaire, walking had a MET of 3.3, moderate physical activity had a MET of 4, and intense physical activity had a MET of 8. The amount of walking (MET × 1 minute × day) should be combined with the amount of moderate physical activity (MET × 1 minute × day) and the amount of intense physical activity (MET × 1 minute × day) in the past week to calculate the total amount of physical activity per week [38]. Physical activity levels of less than 600 are classified as low physical activity, between 600–3000 as moderate physical activity, and more than 3000 as high physical activity [39]. The validity and reliability of the IPAQ in Iran were assessed by Moghadam and colleagues, with its content validity index (CVI) and content validity ratio (CVR) being 0.85 and 0.77, respectively, which indicate the validity of the content. Cronbach's alpha coefficient of 0.7 shows good internal consistency for this tool, and Spearman's correlation coefficient of 0.9 shows its acceptable reliability [40].

#### 2.11.3. 24-Hour Dietary Recall Questionnaire

The 24-hour dietary recall questionnaire was used to assess food intake. In this questionnaire, to evaluate the diet, the mentioned values of each food were converted to grams using a home scale [41, 42]. Then the amounts of nutrients were entered into Nutrathionist 4 (*N*4) software, and the obtained values were analyzed by SPSS software. The validity and reliability of this questionnaire were confirmed in the study of Isfahani and colleagues. The correlation coefficient of this tool was 0.51 for men and 0.59 for women after adjusting the effect of age and energy intake, which indicated its acceptable validity and relative reliability [43].

### 2.12. Data Analysis

Data were analyzed by SPSS software version 22, using descriptive statistics (frequency distribution tables and mean and standard deviation). Paired *t*-test was used to compare the scores of each group before and after the intervention, and one-way analysis of variance with repeated measures (ANOVA), Kruskal–Wallis statistical test, Chi-square, and covariance with adjustment of confounding variable were used to compare the mean scores between the groups before and after the intervention. A significant level of 0.05 was considered in all tests.

### 2.13. Ethical Considerations

The Research Ethics Committee approved this research of the Lorestan University of Medical Sciences with the code “IR.LUMS.REC.1396.399.” Patients were also randomly assigned to one of the control or intervention groups. The treating physician prescribed routine lipid-lowering drugs, and patients were not deprived of their routine treatment. In addition, the confidentiality of individuals' information, the possibility of withdrawing from the study, making the study results available to patients, and financing the project were other ethical considerations that were observed in this study.

## 3. Results

There was no statistically significant difference between the study groups regarding gender, marital status, place of residence, and education level, and the groups were homogenous (Table1). Also, according to the chi-square test, the difference in the frequency distribution of underlying diseases among the study groups was not statistically significant (Table 2).

The one-way analysis of variance showed no significant difference in mean BMI (*P*=0.676) and mean age (*P*=0.253), Amount of energy, carbohydrates, fats, CHOL, saturated fatty acids, unsaturated fatty acids, sodium, potassium, calcium, phosphorus, fiber, soluble fiber, insoluble fiber, crude fiber and fermented fiber received by patients in three groups before the intervention (*P* > 0.05). The results of the Kruskal-Wallis statistical test also showed no statistically significant difference between the 2-gram sumac, 3-gram sumac, and placebo groups in terms of physical activity (*P* > 0.05). Also, according to the chi-square test, the difference in the intensity of physical activity between the three groups was not statistically significant (*P* > 0.05).

Based on the results of repeated measures analysis, the difference in total serum CHOL levels in each group over time was not statistically significant. However, there was a slight change in CHOL level in the 2 g sumac group (time effect) (*P*=0.1). Also, the difference in total serum CHOL levels between the groups in each period (group effect) (*P*=0.299), as well as time-group interaction, which is the difference in mean serum CHOL between groups over time, was not statistically significant (*P*=0.969) (Table 3).

According to the results of repeated measures analysis, the difference in serum LDL levels in each group was significant over time (time effect) (*P* < 0.001). But the difference in serum LDL levels between groups in each period (group effect) (*P*=0.525) and also between groups over time (time-group interaction) was not statistically significant (*P*=0.988) (Table 3).

According to Table 3, the changes in the mean HDL levels in each group over time were not statistically significant. Also, the mean HDL levels between the beginning and end of the sixth and twelfth week were not statistically significant (time effect) (*P*=0.85), although a slight change was observed. Also, between groups in each of the periods before the intervention, six weeks after the intervention, and 12 weeks after intervention (*P*=0.851), as well as the time-group interaction effect, which is the difference in mean HDL serum levels between groups over time was not statistically significant (*P*=0.136).

According to the results of Table 3, the mean changes in serum TG levels in each group over time, at the beginning and end of the sixth and twelfth week were not statistically significant (time effect) (*P*=0.059), although slight changes were observed. Also, the difference between groups in each period (group effect) (*P*=0.459) and time-group interaction was not significant. In the sense that no statistically significant difference was observed over time between serum TG levels in the study groups (time-group interaction effect) (*P*=0.875).

Based on repeated measures analysis, BMI changes in each of the studied groups before and after the intervention were not statistically significant (*P* > 0.05) (Table 4).

## 4. Discussion

According to the results, following the consumption of sumac in the study groups, there was no significant difference between BMI and serum lipid level changes in the groups receiving 2-gram sumac, 3-gram sumac, and placebo over time. However, a significant decrease was observed in serum LDL levels in each group over time (time effect).

In the study of Haj Mohammadi et al., which examined the effect of sumac on 80 patients with hyperlipidemia, no significant difference was observed in LDL levels [44]. The study of Raufi et al., which is in line with the current study, showed that the mean reduction of LDL level in the intervention group was higher than in the control group. Still, this difference was not statistically significant [19]. In addition, the results of a study by Akbari Fakhrabadi et al. also indicated no significant difference in the LDL level between the intervention and control groups [45]. However, some studies have shown that the water-soluble part of sumac acts as a noncompetitive inhibitor of xanthine oxidase and the collector of superoxide radicals, which prevents the increase of serum CHOL. The insignificant effect of sumac on LDL levels in these studies is probably related to the method of consumption and the dosage used, which has not been able to increase the antioxidant capacity. Also, the isoflavones in sumac may not have been sufficient to increase the activity of LDL receptors, which lower CHOL levels [46, 47]. In a study by Rohi Borougeni et al., the researchers concluded that serum lipid levels could be effectively reduced by using sumac, especially in combination with antilipid drugs [48]. Also, contrary to the present study, the findings of a survey by Setorki et al. indicated that the consumption of sumac powder significantly decreased LDL CHOL levels in New Zealander male rabbits. The emulsifying property of sumac originated from the composition of colic acid causes CHOL absorption through its inhibitory effect on the liver's 7-hydroxylase CHOL enzyme.

On the other hand, isoflavones in sumac reduce CHOL levels by increasing the activity of LDL receptors and LDL catabolism in the liver [47]. In addition, the results of a triple-blind clinical trial study by Sabzghabaee et al. showed that taking 500 mg of sumac fruit capsule three times a day significantly altered the LDL levels of obese adolescents with dyslipidemia in the sumac group [49]. Since both doses and the form of the drug were different in the above study, the drug's effectiveness was also significant. However, in our study, 2 and 3 grams of sumac fruit were used once a day as sachets of sumac fruit which has probably influenced the results of this study. Also, the study of Anwar et al. showed that the methanolic extract of sumac significantly redacted LDL and VLDL cholesterol levels in diabetic rats [46]. In this regard, the results of other studies have shown that flavonoid compounds in sumac extract have been effective in reducing LDL oxidation [50–52].

The results of this study also showed that long-term consumption of sumac reduces serum TG levels, but there was no significant change between the groups in each of the periods and over time. These findings are similar to the results of a systematic review and meta-analysis by Akbari Fakhrabadi et al., which examined the effect of sumac on blood lipids [45]. It is possible that the low consumption of sumac in these studies has prevented it from reducing the negative impacts of the peroxidation of blood lipids. But the results of another study by Sabzghabaee et al. showed that TG was significantly altered in the group receiving sumac [49]. Also, contrary to the results of this study, Mir Mohammadi et al. investigated the serum biochemical factors resulting from the consumption of 5 g and 10 g of sumac for ten days on 54 adult male dogs. Their study showed that TG was significantly reduced [30]. Soltani et al. also showed that the mean TG level in mice fed with a fatty diet along with sumac was considerably higher than in the control group. This may be because sumac has antioxidant properties. Antioxidants are factors that can prevent the peroxidation of fats [53]. The results of studies indicate that sumac powder can be effective on gastrointestinal microorganisms by preventing the major reabsorption of bile salts in the presence of specific microorganisms such as *Bacillus subtleties* and *Bacillus licheniformis*. On the other hand, these organisms can synthesize the enzymes esterase (which converts fatty acids in a different form from the structure of TG in the intestine) and lipase (which reduces the absorption of TG) [44, 54].

In addition, the results of the present study showed that total serum CHOL levels in HD patients in each group over time (time effect) and between groups did not change significantly in each period (group effect) and over time (time-group interaction). The study of Akbari Fakhrabadi et al. also showed no significant difference between intervention and control groups [45]. But the study of Anwar et al. showed that sumac significantly reduced TG, LDL levels, and VLDL cholesterol levels [46]. The results obtained in the present study may be due to the low dose of sumac used in patients due to the patient's weight, which indicates the need for further studies in this area. In addition, the results of a survey by Setorki et al. that examined the effect of sumac with CHOL-rich foods on oxidative stress factors that affect atherosclerosis, including glucose, lipid profile, apolipoprotein *B*, nitrate, nitrite, fibrinogen, seven-factor, and liver enzymes in rabbits are also not in line with the results of this study [47]. In this regard, the increase in oxidative stress and lipid peroxidation in renal patients has been stated to be the decrease in plasma levels of various antioxidants. Sumac, with its antioxidant properties, prevents the peroxidation of fats. In the study of Soltani et al., which investigated the effect of sumac extract on blood lipid profile in white rats, the mean blood CHOL in the intervention group was significantly different than the control group [53] which is inconsistent with the findings of our study. Since sumac has a significant amount of water-soluble tannin, it plays an antioxidant role. Also, the phenolic compounds such as phenolic acids, flavonols, and anthocyanins in sumac are a rich source of antioxidants, and a factor in lowering serum CHOL could act as a prophylactic [55]. Until now, few studies have been conducted on the effect of sumac on reducing CHOL levels in HD patients. Also, the present study was performed on a small number of patients. Therefore, more studies with larger sample sizes and more protracted intervention and follow-up periods are recommended on this topic.

The statistical analysis results showed that time did not significantly affect the mean level of HDL. However, in the 2 g sumac group, HDL cholesterol levels were slightly increased between the beginning and end of the sixth and twelfth weeks. However, no significant change in serum HDL levels was observed between the groups at any period and over time. This study revealed that after adjusting the effect of diet and physical activity, the highest and lowest increase in serum HDL levels were observed in the 2 g sumac and placebo groups, respectively, from the sixth to the twelfth week. However, these changes were significant. The study of Salimi et al., which examined the effect of sumac extract on blood glucose and serum lipids in diabetic rats, showed that changes in serum HDL levels were not significant in the groups treated with the sumac extract compared to the control group [29]. Also, in the study of Soltani et al., which examined the effect of sumac extract on blood lipid profile in rats, the mean levels of HDL and LDL did not show a significant change. Due to its phenolic compounds such as phenolic acids, flavonols, and anthocyanins, sumac fruit can act as a rich source of antioxidants and a factor in lowering blood sugar and serum CHOL. Quercetin can also act as a hypolipidemic agent, and probably, one of the reasons for the reduction of serum lipids by sumac is related to this compound [53]. In the study of Sabzeqbaei et al., the clinical effects of sumac fruit on dyslipidemia and HDL cholesterol levels in the intervention and control groups were not significant [49]. The results of a study by Akbari Fakhrabadi et al. also showed no significant difference between the intervention and control groups in terms of HDL levels [45]. Accordingly, the use of sumac extract in higher doses and as prophylaxis is recommended. Haj Mohammadi et al. study also showed that the mean level of HDL cholesterol in the sumac group increased significantly compared to the placebo group [44]. Also, the study of Anwar et al. showed that treatment with sumac significantly increased HDL cholesterol levels, and these findings confirm the usefulness of sumac. Low HDL cholesterol is a risk factor for cardiovascular disease. HDL cholesterol effectively protects arteries against atherosclerosis by removing CHOL from peripheral tissues. In addition, HDL cholesterol has a protective role in the pathogenesis of atherosclerosis by inhibiting the oxidation of lipoproteins such as LDL cholesterol [46].

The present study results showed that sumac fruit has no significant effect on patients' BMI. In contrast, the results of Haj Mohammadi et al. study showed that the consumption of sumac has reduced obesity and its complications [44]. Also, in the study of Sabzeqbaei et al., the BMI of patients with hyperlipidemia in the sumac group was one gram lower than the placebo group [49]. Sumac fruit supplements in these two studies had a significant effect on increasing HDL cholesterol in patients. The phenolic and flavonoid compounds in the sumac plant probably enhance the activity of antioxidant enzymes such as superoxide dismutase and catalase and counteract the damaging effects of free radicals from fatty foods, which ultimately cause weight loss. Achieving these results in our study is probably due to the low intake of sumac fruit due to the weight and specific conditions of the dialysis patients. Therefore, it is suggested to increase its dose in future studies.

In general, in this study, consumption of sumac fruit in the studied groups significantly decreased serum LDL levels over time. However, no significant change was observed in serum LDL levels between the groups in each period and over time. According to research, the water-soluble part of sumac inhibits the enzyme xanthine oxidase and collects superoxide radicals in the body, preventing CHOL increases. However, the lack of effect of sumac on LDL levels between groups is probably related to the method of consumption and the dose consumed, which could increase the antioxidant capacity. Also, the isoflavones in sumac might not have been high enough to increase the activity of LDL receptors, which lowers CHOL levels. The strengths of this study include its human samples that consisted of patients with CRF, the use of two different doses, and the existence of a control group.

This research had some limitations. Although the researchers tried to implement the research protocol by continuously monitoring the patients, some patients might not have followed the protocols. Patients might have taken medication, eaten food, or done strenuous activities without telling us which have affected lipid and BMI levels but such things have not been reported. Due to the inflexibility of the research environment, it was impossible to match the type of dialysis machine and the type of coil used for the patients. Other limitations were the impossibility of selecting a large number of samples as well as the inability of assessing the dry weight of HD patients.

## 5. Conclusion

Consumption of sumac fruit decreased LDL levels over time. However, changes in BMI and serum lipids levels were not significant in patients undergoing HD due to the minimum dose of sumac used in this study regardless of dry weight [56]. Also, there is the possibility that prescribing sumac fruit in the form of medicine will be more accepted by patients rather than testing sumac itself. It is suggested that further studies are needed to be done on HD patients and other high-risk groups with regard to determine the effective dose of sumac and any dose increase should take toxicity into account and consider larger sample size and longer intervention and follow-up times.

## Figures and Tables

**Table 1:** Demographic characteristics of the studied groups.

Demographic variable		Groups			*P* value
		2 g sumac	3 g sumac	Placebo	
		Frequency (%)			
Gender	Man	17 (50)	25 (73.5)	18 (48.6)	0.063
	Female	17 (50)	9 (26.5)	19 (51.4)	
	Total	34 (100)	34 (100)	37 (100)	

Marital status	Single	1 (2.9)	2 (5.9)	2 (5.4)	0.832
	Married	32 (94.1)	31 (91.2)	35 (94.6)	
	Other cases	1 (2.9)	1 (2.9)	0 (0)	
	Total	34 (100)	34 (100)	37 (100)	

Place of residence	City	25 (73.5)	30 (88.2)	32 (86.5)	0.21
	Village	9 (26.5)	4 (11.8)	5 (13.5)	
	Total	34 (100)	34 (100)	37 (100)	

Education level	Illiterate	19 (55.9)	20 (58.8)	19 (51.4)	0.859
	Tips and less	9 (26.5)	9 (26.5)	11 (29.7)	
	High school and diploma	3 (8.8)	3 (8.8)	6 (16.2)	
	University	3 (8.8)	2 (5.9)	1 (2.7)	
	Total	17 (50)	34 (100)	37 (100)	

**Table 2:** Frequency distribution of underlying diseases in study mode groups.

Group		Placebo	3 g sumac	2 g sumac	*P* value
		Frequency (%)			
History of hypertension	Yes	31 (91.2)	28 (82.4)	28 (75.7)	0.222
	No	3 (8.8)	6 (17.6)	9 (24.3)	
	Total	34 (100)	34 (100)	37 (100)	

History of diabetes mellitus	Yes	14 (41.2)	15 (44.1)	11 (29.7)	0.415
	No	20 (58.8)	19 (55.9)	26 (70.3)	
	Total	34 (100)	34 (100)	37 (100)	

History of total cholesterol increase	Yes	7 (20.6)	6 (17.6)	3 (8.1)	0.307
	No	27 (79.4)	28 (82.4)	34 (91.9)	
	Total	34 (100)	34 (100)	37 (100)	

History of TG elevation	Yes	4 (11.8)	5 (14.7)	3 (8.1)	0.681
	No	30 (88.2)	29 (85.3)	34 (91.9)	
	Total	34 (100)	34 (100)	37 (100)	

History of hypoglycaemia	Yes	10 (29.4)	11 (32.4)	14 (37.8)	0.745
	No	24 (70.6)	23 (67.6)	23 (62.2)	
	Total	34 (100)	34 (100)	37 (100)	

History of rheumatic diseases	Yes	4 (11.8)	4 (11.8)	1 (2.7)	0.285
	No	30 (88.2)	30 (88.2)	36 (97.3)	
	Total	34 (100)	34 (100)	37 (100)	

History of gout	Yes	2 (5.9)	1 (2.9)	1 (2.7)	0.744
	No	32 (94.1)	33 (97.1)	36 (97.3)	
	Total	34 (100)	34 (100)	37 (100)	

TG: triglyceride. ^*∗*^*P* < 0.05 for chi-square test.

**Table 3:** Comparison of the mean and standard deviation of changes in CHOL, LDL, HDL, and TG levels in study groups over time.

Variable	Groups	Time			Time effect	Group effect	Time-groupinteraction
		Before *M* ± SD	Six weeks after *M* ± SD	12 weeks after *M* ± SD			
CHOL	2 g sumac	128.6 ± 28.6	122 ± 29.2	127.9 ± 33.1	*F* = 2.366 *P*=0.1	*F* = 1.22 *P*=0.299	*F* = 0.12 *P*=0.969
	3 g sumac	121.9 ± 30.7	118.8 ± 27.5	123.4 ± 262			
	Placebo	132.9 ± 39.1	128.3 ± 41.6	136.8 ± 574			

LDL	2 g sumac	74.3 ± 25.9	67.7 ± 23.5	72.5 ± 22.8	*F* = 7.638 *P* < 0.001	*F* = 0.648 *P*=0.525	*F* = 0.082 *P*=0.998
	3 g sumac	74.3 ± 23.8	63.2 ± 17.9	69.7 ± 20.8			
	Placebo	76.5 ± 25.2	68.8 ± 27.9	76.1 ± 27.9			

HDL	2 g sumac	32.4 ± 7.9	33.4 ± 9.1	35 ± 8.9	*F* = 0.137 *P*=0.85	*F* = 0.161 *P*=0.851	*F* = 1.807 *P*=0.136
	3 g sumac	34.3 ± 14.2	35 ± 12.9	33.9 ± 17.3			
	Placebo	39.6 ± 12.7	33.7 ± 11.9	33.8 ± 8.7			

TG	2 g sumac	104.5 ± 50.7	111.2 ± 77.4	93.7 ± 57.4	*F* = 3.049 *P*=0.059	*F* = 0.784 *P*=0.459	*F* = 0.259 *P*=0.875
	3 g sumac	109.9 ± 77.9	105.5 ± 55.7	92.3 ± 53.2			
	Placebo	122.3 ± 87.7	119.6 ± 66.3	112.5 ± 77			

CHOL: total cholesterol; LDL: low-density lipoprotein; HDL: high-density lipoprotein; TG: triglyceride. *P* < 0.05 for repeated measures analysis (ANOVA).

**Table 4:** Comparison of changes in the mean and standard deviation of BMI in HD patients over time.

Group	Time			Time effect	Group effect	Group-time interaction
	Before intervention	Six weeks after the intervention	12 weeks after the intervention			
	*M* ± SD BMI	*M* ± SD BMI	*M* ± SD BMI			
2 g sumac	23.6 ± 4.4	23.3 ± 4.5	23.6 ± 4.2	*F* = 1.155 *P*=0.317	*F* = 0.333 *P*=0.718	*F* = 0.677 *P*=0.608
3 g sumac	24 ± 5.3	23.8 ± 5.4	23.9 ± 5.4			
Placebo	23.1 ± 3.7	23.1 ± 3.8	23 ± 3.9			

BMI: body mass index. ^*∗*^*P* < 0.05 for repeated measures analysis (ANOVA).

## Data Availability

Not provided.
